# Swedish Population Substructure Revealed by Genome-Wide Single Nucleotide Polymorphism Data

**DOI:** 10.1371/journal.pone.0016747

**Published:** 2011-02-09

**Authors:** Elina Salmela, Tuuli Lappalainen, Jianjun Liu, Pertti Sistonen, Peter M. Andersen, Stefan Schreiber, Marja-Liisa Savontaus, Kamila Czene, Päivi Lahermo, Per Hall, Juha Kere

**Affiliations:** 1 Institute for Molecular Medicine Finland (FIMM), University of Helsinki, Helsinki, Finland; 2 Department of Medical Genetics, University of Helsinki, Helsinki, Finland; 3 Folkhälsan Institute of Genetics, Helsinki, Finland; 4 Department of Genetic Medicine and Development, University of Geneva Medical School, Geneva, Switzerland; 5 Human Genetics, Genome Institute of Singapore, Singapore, Singapore; 6 Finnish Red Cross Blood Transfusion Service, Helsinki, Finland; 7 Department of Neurology, Umeå University Hospital, University of Umeå, Umeå, Sweden; 8 Department of General Internal Medicine, Institute for Clinical Molecular Biology, Christian-Albrechts-University, Kiel, Germany; 9 Department of Medical Genetics, University of Turku, Turku, Finland; 10 Department of Medical Epidemiology and Biostatistics, Karolinska Institutet, Stockholm, Sweden; 11 Department of Biosciences and Nutrition, Karolinska Institutet, Huddinge, Sweden; 12 Clinical Research Centre, Karolinska University Hospital, Huddinge, Sweden; Erasmus University Medical Center, Netherlands

## Abstract

The use of genome-wide single nucleotide polymorphism (SNP) data has recently proven useful in the study of human population structure. We have studied the internal genetic structure of the Swedish population using more than 350,000 SNPs from 1525 Swedes from all over the country genotyped on the Illumina HumanHap550 array. We have also compared them to 3212 worldwide reference samples, including Finns, northern Germans, British and Russians, based on the more than 29,000 SNPs that overlap between the Illumina and Affymetrix 250K Sty arrays. The Swedes - especially southern Swedes - were genetically close to the Germans and British, while their genetic distance to Finns was substantially longer. The overall structure within Sweden appeared clinal, and the substructure in the southern and middle parts was subtle. In contrast, the northern part of Sweden, Norrland, exhibited pronounced genetic differences both within the area and relative to the rest of the country. These distinctive genetic features of Norrland probably result mainly from isolation by distance and genetic drift caused by low population density. The internal structure within Sweden (F_ST_ = 0.0005 between provinces) was stronger than that in many Central European populations, although smaller than what has been observed for instance in Finland; importantly, it is of the magnitude that may hamper association studies with a moderate number of markers if cases and controls are not properly matched geographically. Overall, our results underline the potential of genome-wide data in analyzing substructure in populations that might otherwise appear relatively homogeneous, such as the Swedes.

## Introduction

The recent flood of genome-wide association studies (GWAS) for common diseases has created an upsurge also in studies of population structure based on genome-wide autosomal single nucleotide polymorphism (SNP) array data. This is not only due to the availability of these novel datasets but also due to an increased interest into population structure as a potential confounding factor in the association studies. As a result, this new type of data has already complemented the ones classically used in population genetics. Several studies have shown a general correspondence between genetic and geographic distances within Europe [1]–[3]. Population substructure has also been studied in detail in many European populations, e.g. in Finns [4], [5], Estonians [6], and British [7]. In this paper we study the genetic structure within the Northern European population of Sweden using data from more than 350,000 SNPs genotyped in 1525 Swedes and also compare them to reference samples from several of the neighboring populations.

The first inhabitants to the area of present-day Sweden came after the ice age from Central Europe. For millennia, the country was sparsely inhabited by hunter-gatherer populations until the slow adoption of agriculture and ceramics that began around 4000 BC in southern Sweden [8]. While the southern parts of the country developed strong contacts with the Germanic culture, the north associated to Finland and Karelia with a common culture covering the entire northern Fennoscandia. This culture has sometimes been suggested to be ancestral to the indigenous Sami population still inhabiting the area. Sweden was not united under one ruler until the 11th century, and the traditional division to the southern Götaland, central Svealand, and northern Norrland is still widely known despite lacking any official status. There have been long-standing contacts with the neighboring populations, with Norwegian influence in western Sweden, Danish in the south, and Finnish in the north [9], [10]. The population density has been highest in Southern and Central Sweden, while in Norrland the population is centered on the eastern coast and in river valleys whereas the mountaineous regions in the northwest are largely uninhabited.

Genetically the Swedes have appeared relatively similar to their neighboring populations - for example the Norwegians, Danish, Germans, Dutch and British - both in a classical study based on a small number of autosomal markers [11] and in the recent genome-wide studies [1]-[6], [12]. Similar patterns of a close relationship with neighboring populations have been observed in the Y-chromosomal and mitochondrial DNA (mtDNA) variation [13]. In contrast, the Finns seem to be an exception to this rule: they do not appear genetically very close to the Swedes although they are geographically nearby. However, the Finns tend to show inflated genetic distances relative to the European populations in general [1], [4], [6], not only relative to the Swedes.

The internal genetic structure of the Swedish population has been mostly studied with the Y chromosome and mtDNA. These studies have shown haplogroup frequency differences within the country [14], [15] that are mostly clinal but also reflect the effects of local genetic drift and reveal signs of influence from neighboring populations into respective parts of the country [15]. On the other hand, a study with 34 unlinked autosomal SNPs found little population structure within Sweden [16]. The river valleys in Northern Sweden have shown genetic differentiation in terms of the frequency of protein markers [17]. Studies of ancient DNA have shown a genetic discontinuity between the Neolithic inhabitants of the southern part of Sweden (ca. 3000 BC) and the current Swedish population [18].

In this study, we have analyzed the current autosomal population structure within Sweden using 1525 individuals genotyped on the Illumina HumanHap550 SNP array, and compared the Swedes also to Finns, Germans, Russians and other reference populations. We observed that the Southern Swedes were genetically close to northern Central Europeans and exhibited subtle genetic substructure, whereas the northern part of Sweden, Norrland, clearly differed from the rest of the country and showed significant internal structure.

## Results

### Swedes relative to neighboring populations

**Figure 1:**
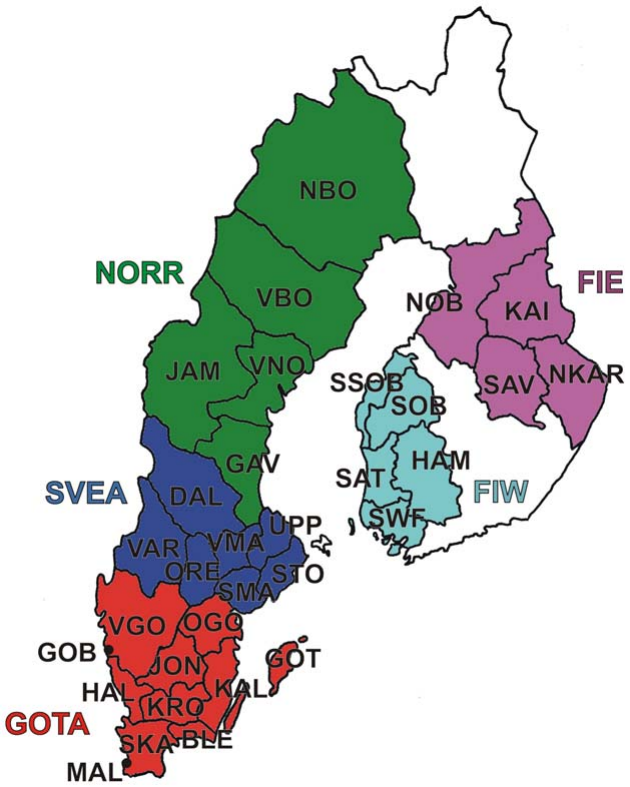
A map of the studied provinces and regions of Sweden and Finland. Full population names and their sample sizes are given in Table 1 and Table S1.

**Figure 2:**
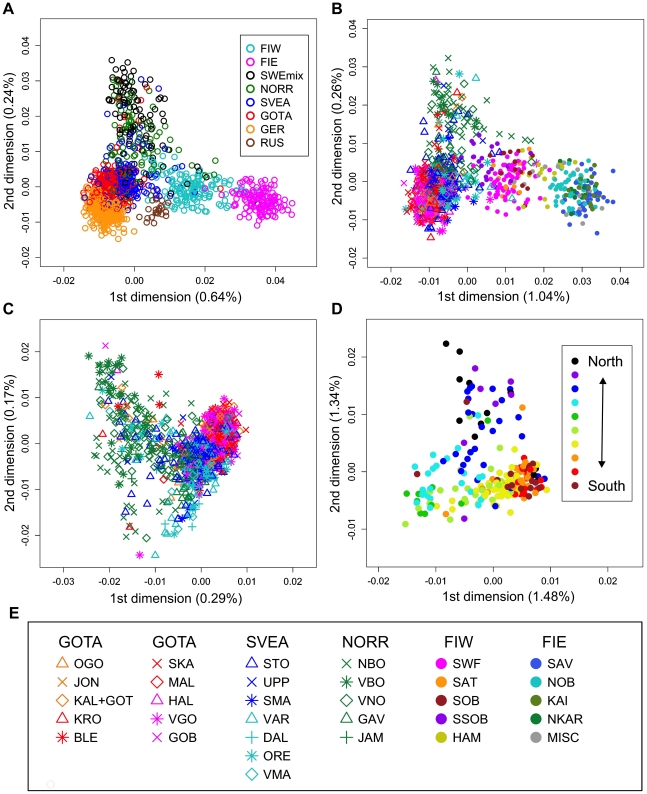
Multidimensional scaling plots of genetic distances between individuals. Identity by state (IBS) distances in Northern Europe (a), Sweden and Finland (b), Sweden (c) and Norrland (d), with the legend for panels (b) and (c) in (e). The axis labels show the proportion of variance explained by the axis. Abbreviations as in Table 1 and Table S1. In (d), the colouring of individuals represents one of the ten major river valleys of Norrland, from north to south. See also Figure S1 for animated three-dimensional versions of (a) and (b).

**Figure 3:**
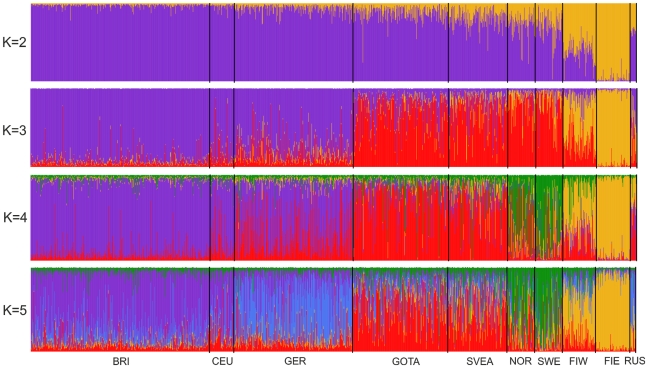
Clustering of North European individuals by the Structure software. Each individual is represented by a thin vertical line, and their proportions of ancestry in each of the K inferred clusters (from 2 to 5) are denoted by colors. Abbreviations as in Table 1.

We used genome-wide SNP genotypes of 1525 Swedes and 3212 worldwide reference individuals to study the autosomal population structure within Sweden and relative to neighboring populations (Fig. 1, Table 1, Table S1; see Methods for details of the datasets). A multidimensional scaling (MDS) plot of identity by state (IBS) distances (pairwise proportions of alleles not identical by state) in Northern Europe (Fig. 2a) showed clustering of individuals primarily according to their area of origin, and revealed a triangular pattern with Northern Swedes and Eastern Finns in the two furthest corners; the third dimension (Fig. S1) further differentiated Germany from Southern Sweden (Svealand and Götaland). There was an overall correspondence between geographic and genetic distances, with the exception that Northern Swedes and Eastern Finns exhibited longer genetic distances than their geographic location would imply. Focusing further, the MDS plot of Swedes and Finns colored according to the province of origin (Fig. 2b, Fig. S1) exhibited a similar triangular pattern, with Northern Sweden, Southern Sweden (Svealand and Götaland) and Eastern Finland spanning the corners, and showed a fairly high degree of overlap between provinces, especially in Southern Sweden. Of the Swedes, Norrland and Svealand individuals were closest to Finns, and the Finns who had closest affinity to the Swedes were mainly Swedish-speaking Ostrobothnians (SSOB). Interestingly, the neighboring Swedish and Finnish provinces in the north, Norrbotten (NBO) and Northern Ostrobothnia (NOB), did not appear very close in the MDS plot; instead, Norrbotten seemed to show closer affinity to Western Finland. A Structure analysis of Europeans (Fig. 3) showed successive clusters (two to five) dominated by Eastern Finns, Swedes, Northern Swedes and Germans, respectively. The sixth and seventh clusters (not shown) did not bring out further differences. The likelihoods of clusterings appeared approximately equal (Fig. S2); using a specific statistic [19], the most likely numbers of clusters were 2 or 6.

**Table 1:** Names, abbreviations and sample sizes of the main study populations.

Country	Region	Abbreviation	Sample size in Dataset 2	Sample size in Dataset 3
Sweden				
	Norrland	NORR	115	237
	Svealand	SVEA	245	545
	Götaland	GOTA	395	743
	unknown	SWEmix	113	0
Finland				
	Western Finland	FIW	141	0
	Eastern Finland	FIE	139	0
Germany		GER	492	0
Russia		RUS	25	0
Great Britain		BRI	740	0
CEPH^a^		CEU	101	0

The corresponding information for the Swedish and Finnish provinces is given in Table S1.

a Utah residents with Northern and Western European ancestry from the CEPH collection.

**Figure 4:**
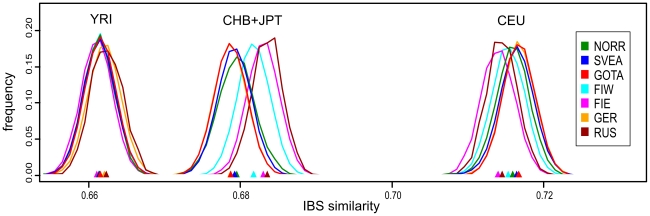
Distributions of pairwise identities by state between North European populations and four HapMap populations. Each curve represents the IBS similarities of all pairs of individuals where one individual is from the HapMap population in question and one from the population indicated by the color of the curve. The location of distribution medians is denoted by triangles of corresponding color. All distributions with CEU differed significantly (p<0.015) except Eastern Finland vs. Russia, Götaland vs. Germany, and Svealand vs. Germany. All distributions with CHB+JPT differed significantly (p<0.002) except Eastern Finland vs. Russia, Götaland vs. Germany, and Svealand vs. Norrland. In the comparison with YRI, Germany and Russia differed significantly from all populations except each other, and Eastern Finland from Götaland (p<0.027 for each). These p values have been Bonferroni-corrected. Abbreviations: Yoruba from Ibadan, Nigeria (YRI, n = 105); Han Chinese from Beijing, China (CHB, n = 78); and Japanese from Tokyo, Japan (JPT, n = 84); other abbreviations as in Table 1.

In analyses with predefined population divisions, the F_ST_ distances between European populations (Table S2, Fig. S3a) showed a pattern mostly corresponding to geographic distances, with the exceptions of Eastern Finns (and to a certain degree also Western Finns), Basques and Sardinians showing longer genetic than geographic distances. The overall levels of allele frequency differences between North European populations showed a similar pattern (Table 2), with Eastern Finns differing the most, and Swedes - especially in Svealand and Götaland - being relatively close to Central Europeans (Germans and British). The IBS distributions between Northern Europeans and HapMap populations (Fig. 4) showed that Götaland and Germany were most similar and Eastern Finns and Russians least similar to HapMap CEU, while in the comparison with HapMap CHB and JPT, the opposite order emerged (Bonferroni-corrected p<0.015 for all distribution pairs, except Götaland vs. Germany and Eastern Finland vs. Russia nonsignificant with respect to both HapMap populations, Germany vs. Svealand with CEU, and Norrland vs. Svealand with CHB and JPT). However, a very different pattern was observed when comparing with the Russians (Fig. S4a): Norrland and Eastern Finland showed the least similarity, Svealand and Götaland an intermediate amount, and Germany and especially Western Finland the most (Bonferroni-corrected p<0.031 for Western Finland vs. all other populations except Germany, and for Germany vs. Norrland and Eastern Finland). The F_ST_ distances between the Swedish and Finnish provinces (Table S3, Fig. S3b) repeated the features seen in the MDS, with the Swedish-speaking Finns (SSOB) being closest to Sweden and Northern Ostrobothnia (NOB) not very close to northern Norrland; furthermore, the distances between the Swedish provinces were generally smaller than those between the Finnish provinces.

**Table 2:** Degree of allele frequency differences between population pairs.

λ	FIE	FIW	NORR	SVEA	GOTA	GER	BRI
FIE	1.00	1.71	2.59	2.62	2.91	3.08	3.30
FIW	1.71	1.00	1.56	1.52	1.70	1.82	2.05
NORR	2.59	1.56	1.00	1.12	1.20	1.36	1.46
SVEA	2.62	1.52	1.12	1.00	1.03	1.16	1.28
GOTA	2.91	1.70	1.20	1.03	1.00	1.13	1.21
GER	3.08	1.82	1.36	1.16	1.13	1.00	1.11
BRI	3.30	2.05	1.46	1.28	1.21	1.11	1.00

Measured as the overdispersion factor (λ) of observed vs. expected chi-square statistics; λ = 1 indicates no difference. Abbreviations as in Table 1.

### Variation within Sweden

**Figure 5:**
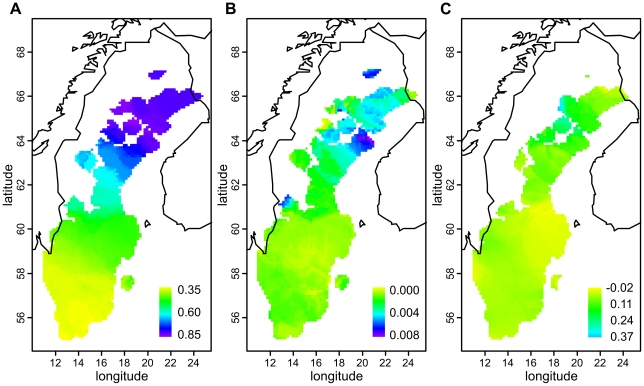
Local genetic variation within Sweden. The colour of each area corresponds to the local value of median ancestry proportion in one of two Structure-inferred clusters (a), median inbreeding coefficient (b) and correlation of genetic and geographic distances (c), calculated in circles with a radius of 150 km and depicted only for those circles that had at least 20 samples (at least 40 in (c)).

The MDS plot of the Swedes alone (Fig. 2C) showed a north-south gradient in the first dimension and a spread between Västerbotten (VBO) and Norrbotten (NBO) in the second, whereas the Southern Swedish samples remained tightly clustered. Again, a fair degree of overlap was seen between the provinces. When MDS was done for Southern Swedes separately (Fig. S5), the first dimension suggested a north-south gradient, and the second dimension a subtle degree of structuring within Götaland. MDS of the Norrland samples alone, with a north-south colouring according to ten major river valleys (Fig. 2D), revealed a loose division into three: northern, middle and southern parts of Norrland; notably, the middle differed in the first dimension and the north only in the second. A Structure analysis discovered two clusters within Sweden (3 clusters were also tested but yielded a lower likelihood); these clusters showed an overall north-south cline in frequency, and ancestry in one of them was especially common in Västerbotten (Fig. 5a, Fig. S6). Similarly, inbreeding (Fig. 5b) showed a cline with stronger inbreeding in the north, strongest in coastal Västerbotten (p<0.0002 for inbreeding differences between the three Swedish regions). The correlation between genetic and geographic distances was significant in Sweden as a whole (r = 0.066, p<0.0001) and stronger in Norrland (r = 0.164) than in Svealand or Götaland (r = 0.011 and r = 0.036, respectively; p<0.0001 for all three regions). Concordantly, a local analysis (Fig. 5c) showed the strongest correlation in the north, especially in Västerbotten.

In terms of F_ST_, the differences between provinces were small but significant within the whole of Sweden as well as within Norrland and Götaland (0.0005, 0.0009 and 0.0002, respectively; p<0.0002 for each) but not within Svealand (p = 0.19). (For comparison, the population structure among the British reference samples was nonsignificant (p = 0.08).) When F_ST_ was analyzed between the three regions and the provinces simultanously, differences both among the regions (F_ST_ = 0.0004) and among the provinces within the regions (F_ST_ = 0.0003) were significant (p<0.0002 for both). Pairwise F_ST_ values between the Swedish provinces (Table S4, Fig. S3c) showed that the two northernmost provinces, Norrbotten (NBO) and Västerbotten (VBO), differed most from the rest of the provinces and also significantly from each other. This was also seen in a Barrier analysis (Fig. S7), where the two first barriers were located in the north. In terms of IBS similarity within the population (Fig. S4b), Eastern Finland differed significantly from all other populations, Norrland from Götaland and Western Finland, and Western Finland from Svealand (Bonferroni-corrected p<0.034 for each); interestingly, the similarity in Norrland was among the lowest. Linkage disequilibrium (LD) (Fig. S8) was stronger in Norrland than in the two other Swedish regions; all three regions showed weaker LD than Eastern and Western Finland but stronger than Germany and Great Britain (p<0.002 for all pairwise comparisons, except Svealand vs. Götaland and Germany vs. Great Britain nonsignificant).

Allele frequencies in Svealand and Götaland appeared very similar (Table 2), but the differences between them and Norrland were of the same magnitude or larger than between Germany and Great Britain. Between Svealand and Götaland, an allele frequency difference with p<0.05 was observed for 5.1% of the SNPs, whereas Norrland differed from Svealand and Götaland for 6.4% and 7.2% of the SNPs, respectively. For comparison, corresponding proportions were 13.4% between Eastern and Western Finland and 5.2% within Britain (Scotland and Northern vs. Eastern and Southeastern areas). However, the small sample size in these comparisons (n = 115 per population) obviously limited the power to detect significant differences: in our largest dataset, 13.1% of the SNPs showed a chi-square p<0.05 between Norrland and Götaland (n = 237 and n = 743, respectively). The SNPs with the largest allele frequency differences between Norrland and the rest of Sweden were relatively scattered across the genome (Fig. S9); while the genes closest to these SNPs showed no systematic enrichment into any Gene Ontology class, nominally significant SNPs were unexpectedly common in the MHC region and in genome areas associated to skin pigment and blood lipid traits (Table S5). However, the latter result remains suggestive, as the analysis did not correct for differing LD patterns across genome areas. The topmost differing SNPs and their closest genes are listed in Table S6, and all SNPs with p<0.001 in Table S7.

## Discussion

In this study, we have used genome-wide SNP data to analyze the population structure within Sweden, and discovered a clinal north-to-south structure that was particularly pronounced in the northern part of the country. The Swedes showed a considerable genetic difference from the neighboring population of Finns, whereas the southern Swedes appeared genetically very close to northern Central Europeans: northern Germans, British, and the HapMap CEU samples.

In population genetic studies, systematic differences in sampling and genotyping are a potential confounder and may inflate the observed population structure. This warrants caution in our study which combines data genotyped on various platforms in several genotyping centers, but it is unlikely to cause major errors since several population comparisons showed little differentiation across datasets. Using combined cases and controls from Sweden is also unlikely to have a substantial effect on our results, because after the exclusion of the most differing SNPs, these sample groups hardly differed, and similar results were obtained also without the cases. Furthermore, technological biases can also be partly alleviated by our choice of analysis methods that are not overly sensitive to small numbers of differing SNPs, e.g. MDS instead of principal component analysis (PCA), and by limiting the analyses to the SNPs that have been genotyped in all the populations, instead of using imputed data.

An issue of bigger concern are the effects of the sampling scheme, both in terms of ancestry ascertainment and geographic distribution. For instance, although we observed a much more subtle internal structure in Sweden than in Finland, it is difficult to estimate to which degree the difference was caused by the differential ascertainment (for place of residence vs. grandparental birthplace, respectively). Nevertheless, the substructure within Sweden was significant, stronger than between Northern and Southern Germany [20] or within Britain (this study), and consistent with earlier studies using Y-chromosomal and mitochondrial DNA markers [14], [15]. The overall proportion of variance explained by the first MDS dimensions was small, reflecting the well-known fact that most of the genetic variation in humans lies between individuals. The effects of differential geographical sampling were also demonstrated: When we compared the Swedish samples from our earlier study that lacked detailed ancestry information [4] to the larger dataset of this study, we observed that the former samples likely originate predominantly from Norrland. Therefore, the relatively large difference between the two datasets (F_ST_ = 0.0012) is likely caused by a difference in the geographical sampling distributions. Notably, the datasets also behaved rather differently in the F_ST_ comparisons to Central Europeans. This nicely illustrates how differences in geographical sampling between studies could affect quite a lot the way that Swedes appear in comparisons with other populations.

The northern part of Sweden, Norrland, showed a particularly strong population structure, which could be explained by genetic drift in this sparsely inhabited region. However, this hypothesis was challenged by the genetic diversity within Norrland that was not consistently reduced: in fact, Norrland showed significantly lower IBS similarity than Götaland. This could suggest the presence of several isolates within Norrland, and indeed, signs of such were detected in the MDS and F_ST_ analyses. Furthermore, LD in Norrland was stronger than in the rest of Sweden. Together, these patterns of variation could be partly explained by migration. While the influence from Finland seemed moderate, at the most, we unfortunately lacked the reference samples necessary for analyzing possible Sami and Norwegian contributions. However, earlier mitochondrial DNA and Y-chromosomal studies have indicated influence from the Sami and/or Finns in Northern Sweden, as well as decreased genetic diversity [14], [15]. A pattern of pronounced genetic differences similar to those in Norrland has been previously observed in the northern parts of Finland [5]. However, Eastern Finland showed a different combination of signs of drift: strong LD and low diversity. These probably stem from the major founder event during the 16th century migration wave [21] that appears to have affected the gene pool more profoundly than subsequent drift within local population isolates. Thus, not all small and drifted populations are alike, and the relevant geographic scale of drift can vary.

Interestingly, the Finnish province genetically closest to Norrland was not the neighboring Northern Ostrobothnia, but the Swedish-speaking Ostrobothnia and Southwestern Finland hundreds of kilometers further south. Although this pattern might first seem surprising, it is consistent with the history of Northern Ostrobothnia, where the current population is largely derived from a 16th-century migration that originated from the province of Southern Savo [21]. The arrival of these genetically distinct [4] eastern migrants may have broken a possible earlier genetic cline along the coasts of Northern Sweden and Western Finland, and despite the later contacts across the border, the following centuries might not have been long enough a time to fully restore the cline.

Among our Finnish sample, genetically closest to Swedes were the Swedish-speaking Finns of coastal Ostrobothnia. This agrees well with the history of the Swedish-speakers, who arrived into the western and southern coastal areas of Finland in the beginning of the second millennium [21]. However, they have obviously experienced a lot of subsequent admixture with the Finnish-speakers, resulting in a subtle difference between them and their closest neighbors; conversely, their genetic distance from the Swedes is of the same magnitude as the largest distances between provinces within Sweden. A similar, intermediate position of the Swedish-speakers has been detected earlier [22], although with differing admixture proportions, probably depending on the choice of reference samples.

In our earlier study [4], we saw that North European populations exhibited differing amounts of IBS similarity to East Asians so that Finns, especially Eastern Finns, were the most similar. Now we have observed the same phenomenon - though in a smaller degree - within Sweden, where Norrland showed the most of East Asian similarity and Götaland the least. This is consistent with earlier Y-chromosomal studies [13]. In strong contrast, however, neither Norrland nor Eastern Finns showed any increase in similarity to the Vologda Russians, and a similar lack of affinity between Finns and Russians can also be seen in separate datasets [6], [13]. Thus, if the current references are representative of Russians in this respect, the observed affinity to Eastern Asia would not be mediated by contacts with Russians but could reflect an ancient eastern influence predating the arrival of Slavic populations to Northeastern Europe in the end of the first millennium [23]. It remains unclear whether the eastern affinity observed in Sweden would date back to the same era, or rather reflect the amount of later Finnish contacts to the area.

Several studies have now shown a general correspondence between geographic and autosomal genetic distances between European populations [1]–[3], and a similar pattern was seen in our data. However, the exact strength of this correspondence seemed to vary substantially: In Southern Sweden and in northern Central Europe, a given genetic distance corresponded to long geographic distances, which would be consistent for example with a scenario of relatively large breeding units and moderate effects of genetic drift balanced by frequent migration. In Northern Sweden, Western Finland and especially in Eastern Finland, similar genetic distances were observed across much shorter geographic distances, suggesting that in these areas, genetic drift may have been a more powerful force shaping the gene pool. Thus, the mere notion of an overall correlation between geographic and genetic distances is insufficient to describe the complexity of the Northern European genetic landscape and its demographic determinants.

Population substructure can be a crucial issue in association studies, where population differences between cases and controls can cause spurious association signals. In GWAS, it is possible to correct for population stratification by using the bulk of data that is not assumed to correlate with the phenotype of interest, but in replication or candidate gene-based association studies that involve a more limited number of markers, such corrections are not possible. The amount of allele frequency differences we detected within Sweden warrants caution when matching controls for cases geographically, especially if individuals with descent from the northern part of Sweden are involved: for example in a study with cases from Norrland and controls from Götaland, a random SNP would have a substantially inflated chance of showing a chi-square p<0.05 due to the population structure alone - even in our moderately sized dataset of less than 1000 individuals, the chance was 13%. As the observed structure within Sweden is mostly caused by random forces such as drift, the differing SNPs are scattered throughout the genome, and there is no means of recognizing them without prior population data. Thus, especially with phenotypes where cases are likely to be geographically clustered, rigorous matching of controls may be needed in order to avoid effects of stratification.

Genome-wide SNP datasets are quickly proving their usefulness in population genetic studies. Firstly, such datasets greatly increase the number of available loci, and they can therefore yield a more balanced picture of the diverse aspects of a population's history than for instance the uniparental markers that comprise only two loci. Secondly, the large number of individuals typically involved in a GWAS improves the resolution of population genetic analyses. Admittedly, GWAS control individuals can lack detailed ancestry information or might not represent populations with particularly interesting ancestry, which may limit their utility for population history studies. Nevertheless, studies such as ours that are based on residence information can uncover the patterns of the current population structure, which are often more important for practical applications, and still provide novel information of the population history in high precision.

## Materials and Methods

### Ethics statement

All the samples were analyzed anonymously. The Swedish, Finnish and German samples were collected with informed consent according to the principles of the Declaration of Helsinki, and their use for population genetics study was approved by the ethics committees of the Karolinska Institute, Umeå University, Finnish Red Cross, and the Kiel Medical Faculty. For details of the collection of the British and worldwide samples, see the references below.

### Genotypes

We used genome-wide SNP genotypes from altogether 1642 Swedes, 280 Finns, 492 Germans, 740 British, and 1587 worldwide population samples to study the genetic structure in Northern Europe, especially within Sweden. The Swedes consisted of 774 case and 755 control females from a breast cancer study [24] who had been ascertained for place of residence in 1993–1995, and 113 male population samples of ethnic Swedes mainly from eastern Sweden but without further geographic information [4]. The Finns were male blood donors, 141 of whom had grandparental birthplaces in Western and 139 in Eastern Finland [4]. The Germans were male and female control samples from the PopGen cohort from Kiel area in Schleswig-Holstein in Northern Germany [25]. The British were male controls of the 1958 birth cohort whose genotype data were kindly provided by the Wellcome Trust Case Control Consortium [26]. Additionally, we used the publicly available genotypes of 860 individuals from Human Genome Diversity Project (HGDP, http://www.stanford.edu/group/morrinst/hgdp.html) [27] and 727 individuals from HapMap phase 3 release 1 (http://hapmap.ncbi.nlm.nih.gov) [28] as worldwide reference samples; in particular, analyses of Northern Europe included 101 CEU samples (Utah residents with Northern and Western European ancestry from the CEPH collection) from HapMap and 25 Russians (from Vologda, ca. 550 km east of Saint Petersburg) from HGDP.

### Quality control and the different datasets

The genotypes used in this study originated from various platforms: the Swedish females from Illumina HumanHap550 (San Diego, CA); the Swedish males, the Finns, the Germans and the British from Affymetrix 250K StyI (Santa Clara, CA); the HGDP samples from Illumina HumanHap650K; and the HapMap samples from Affymetrix 6.0 and Illumina 1M. DNA extraction, genotyping and genotype calling had been done for the datasets separately according to manufacturers' instructions and is described in more detail in the references given above. Quality control (QC) filtering and LD-based pruning were done using Plink version 1.06 (http://pngu.mgh.harvard.edu/purcell/plink/) [29]. Three datasets were formed (Table S8) to maximize the number of SNPs, reference populations, and Swedish samples, respectively. Dataset 1 consists of Illumina-genotyped individuals: HapMap, HGDP, and the Swedish breast cancer controls. QC thresholds were 99% success per individual and 95% success per SNP in each of the three source datasets, HWE p = 10^−7^ for each population (except for HapMap, where the source dataset had been filtered for p = 10^−6^) and minor allele frequency (MAF) 0.05 for the whole dataset. In 119 pairs of individuals with high identity by state (IBS) similarity, the individual with lower genotyping success was excluded; additionally, the whole MKK population of HapMap was excluded due to multiple cases of high IBS relatedness. In Dataset 2, also the Affymetrix genotypes (the Swedes without geographic information, Finns, Germans and British) were added. Only the markers overlapping between the source datasets were used; no imputation was done. QC thresholds were the same as for Dataset 1, except for individual success 97% for the Affymetrix individuals. Nine SNPs were removed due to highly discordant frequencies (chi-square p<10^−10^) between comparable parts of the Affymetrix and Illumina datasets (the two Swedish datasets against each other, and the British vs. HapMap CEU). Dataset 3 consists of the Swedish individuals of Dataset 1 supplemented by Swedish cases (774 cases and 751 controls). In addition to the same QC procedure as for Dataset 1, 41868 SNPs with p<0.1 in a chi-square test of allele frequencies between cases and controls were excluded; Fig. S10 shows the remaining differences. Each of the three datasets was pruned based on LD between markers to form both a pruned and a highly pruned dataset. Table S8 gives the number of individuals and SNPs in each of the datasets, and Table S9 indicates the dataset used in each analysis.

### Geographical coordinates and province divisions for Swedes and Finns

Coordinates for the Swedish individuals were obtained from GeoNames (www.geonames.org) based on both their postal code and county information, and confirmed from Google Maps in case of disparity. Coordinates for the Finnish individuals were based on the grandparental birthplaces, and a mean of these was used as a coordinate for the individual.

The places of residence of the Swedish individuals were distributed throughout the country, approximately reflecting the population density. For frequency-based analyses, the Swedes were divided into 21 provinces according to the administrative divisions (“län”) of Sweden (Table S1). Some provinces were combined to reach sufficient sample sizes. The largest cities (Malmö and Gothenburg) were separated from their surrounding provinces (Skåne and Västra Götaland, respectively). The provinces were further combined according to a traditional division to form three regions: Norrland, Svealand, and Götaland. The resulting sample sizes per province and region are shown in Table S1.

### Analyses

The F_ST_ calculations were done in Arlequin 3.11 [30], using 5000 permutations to calculate the p values. IBS matrices between pairs of individuals were calculated in R 2.7.2 (www.R-project.org) [31] package GenABEL 1.4–2 [32]. Matrices of IBS distance (1-IBS) and F_ST_ were visualized by classical multidimensional scaling (MDS) in R. The proportion of variance explained by each MDS dimension was calculated as the ratio of the respective eigenvalue to the sum of all eigenvalues [33]. The statistical significance of the difference between the IBS distributions was calculated by comparing the observed Mann-Whitney U test statistic to its empirical distribution based on 10.000 permutations of population labels for each pair of populations. Within each population, LD between 5083 randomly chosen SNPs and all other SNPs at less than 500 kb distance from them (altogether 67620 pairs) was calculated using the EM algorithm in Plink. The statistical significance of the differences in LD between populations was evaluated using a Wilcoxon signed rank test (i.e., a paired test) in R. Great-circle distances from R package fields [34] were used as the geographic distances between individuals. The correlation of the geographic and genetic (1-IBS) distance matrices was tested with Mantel test using the R package ade4 version 1.4 [35].

Allele frequency differences in pairs of populations were calculated by a chi-square test (1 degree of freedom) in Plink, and the general degree of inflation (λ) was calculated as the ratio of the means of the smallest 90% of observed vs. expected chi-square test statistics as in [36]. The degree of allele frequency differences between cases and controls was calculated similarly, and visualized with a quantile-quantile plot. To study whether the genetic differences within Sweden would be disproportionately often related to a certain gene function or pathway, we determined for each SNP the closest gene (within 200 kb), and compared the Gene Ontology classifications of the genes for the most differing SNPs (with -log(p) cutoffs 3, 3.5 and 4 resulting in 431, 190 and 87 genes, respectively) to those of the whole dataset using Panther version 7 (http://www.pantherdb.org) [37], [38]. For eight interesting phenotypes, we also checked whether their associated genome areas (those within 200 kb of associated SNPs listed in National Human Genome Research Institute's Catalog of Published Genome-Wide Association Studies (http://www.genome.gov/gwastudies) [39] on 11/16/2010) harbored unexpectedly many SNPs with nominally significant regional allele frequency differences.

Individuals were clustered with Structure 2.2 software [40] using the admixture model and 10,000 burn-ins and iterations. Three separate runs were done for each number of clusters (K). The relative likelihoods of different K were judged from the run probabilities and by visual inspection of the resulting clusters as well as with a specific statistic [19]. Inbreeding coefficients of Swedish individuals relative to the total Swedish sample (in terms of observed vs. expected homozygotes) were calculated in Plink, and the differences between the three regional distributions were tested with a Kruskal-Wallis rank sum test. In addition to comparing regional distributions, inbreeding was analyzed within local geographical units: for a set of grid points on a map, each grid point that had at least 20 samples within a 150 km distance was plotted on the map in a color corresponding to the median inbreeding coefficient of those samples. A similar grid-based visualization was done for the individuals' proportions of Structure-inferred ancestry and for the correlation coefficient of genetic and geographic distances. The latter used, for each grid point, all pairs of individuals within a 150 km distance. The areas of strongest genetic change between Swedish provinces were analyzed using Barrier software v. 2.2 [41] on the residuals of a linear regression of the genetic distance (F_ST_) on the geographic distance (between provinces' average sample locations). In allele frequency difference and LD analyses all populations were sampled to 115 individuals to avoid the effects of sample size. The reported significance levels for the IBS distributions, LD and inbreeding have been Bonferroni-corrected for the number of tests done within each analysis.

## Supporting Information

Figure S1 **Multidimensional scaling plots of genetic distances between individuals in three dimensions.** Identity by state distances in Northern Europe (left), and Sweden and Finland (right). The proportions of variance explained by the three axes are 0.64%, 0.24%, and 0.17% in Northern Europe and 1.04%, 0.26%, and 0.24% in Sweden and Finland, respectively. The animation files can be opened e.g. in most internet browsers. Abbreviations as in Table 1 and Table S1.
(GIF)

Figure S2 **The probabilities of different numbers of clusters (K) in the Structure analysis of Northern Europe.**
(TIF)

Figure S3 **F_ST_ distances visualized by multidimensional scaling.** Pairwise distances between European populations (a), Swedish and Finnish provinces (b), and Swedish provinces (c). The corresponding F_ST_ values can be found in Tables S2-S4. Abbreviations: Toscans in Italy (TSI) from HapMap; French (FRE), French Basque (FRB), North Italian (ITN), Orcadian (ORC), and Sardinian (SAR) from HGDP; Swedes with geographical information (NORR+SVEA+GOTA) (SWE); other abbreviations as in Table 1 and Table S1.
(TIF)

Figure S4 **Distributions of pairwise identities by state (IBS).** IBS distributions between six populations and Russians (a) and within the six populations (b). The location of distribution medians is denoted by triangles of corresponding color. In (a), Western Finland differed significantly from all other populations except Germany, and Germany from Norrland and Eastern Finland (p < 0.031 after a Bonferroni correction). In (b), Eastern Finland differed significantly from all other populations, Norrland from Götaland and Western Finland, and Western Finland from Svealand (p < 0.034 after a Bonferroni correction). Abbreviations as in Table 1.
(TIF)

Figure S5 **Multidimensional scaling plots of genetic distances between individuals in Svealand and Götaland.** The genetic distance used is based on identity by state (IBS). Abbreviations as in Table S1.
(TIF)

Figure S6 **Clustering of Swedish individuals by the Structure software.** Each individual is represented by a thin vertical line, and its proportional ancestry in the two inferred clusters is denoted by colors. The individuals are sorted according to latitude from south to north.
(TIF)

Figure S7 **Six zones of strong genetic change inferred by the Barrier software.** The borders (in red) are numbered in decreasing order of strength, and they are based on F_ST_ distances that have been corrected for the geographical distance between provinces. Note that the F_ST_ values differ significantly from zero only for the first two borders.
(TIF)

Figure S8 **Linkage disequilibrium as a function of distance between markers.** Median D' in overlapping 40 kb windows at 10 kb intervals is plotted for each population using 67620 marker pairs. All distributions differed significantly (p < 0.002) except Germany vs. Great Britain and Svealand vs. Götaland. Abbreviations as in Table 1.
(TIF)

Figure S9 **Genomic locations of SNPs whose allele frequency differs in Norrland.** The p value from a chi-square test of allele frequencies between Norrland (n  =  115) and the rest of Sweden (n  =  635) is indicated for each SNP. The most differing SNPs are listed in Table S6 and Table S7.
(TIF)

Figure S10 **Differences between cases and controls in Dataset 3.** A quantile-quantile plot of observed vs. expected test statistics (in blue) from a chi-square test of allele frequency differences between cases and controls for the SNPs that remain in Dataset 3 after quality control. Lambda denotes the overdispersion factor of observed vs. expected chi-square statistics.
(TIF)

Table S1 **Names, abbreviations and sample sizes for the Swedish and Finnish provinces.**
(XLS)

Table S2 **Pairwise F_ST_ values (multiplied by 10,000) between European populations.**
(XLS)

Table S3 **Pairwise F_ST_ values (multiplied by 10,000) between Swedish and Finnish provinces.**
(XLS)

Table S4 **Pairwise F_ST_ values (multiplied by 10,000) between Swedish provinces.**
(XLS)

Table S5 **Number of SNPs with geographical differentiation within Sweden located in genome areas associated with eight phenotypes.**
(XLS)

Table S6 **The SNPs with largest allele frequency difference between Norrland and the rest of Sweden, and their nearest genes (within 200 kb).**
(XLS)

Table S7 **A list of the SNPs whose frequency differs between Norrland and the rest of Sweden (chi-square p<10^−3^).**
(XLS)

Table S8 **The numbers of SNPs and individuals in the different datasets after quality control.**
(XLS)

Table S9 **The datasets used in each analysis.**
(XLS)
