# Supplementary figures and images for: Swedish Population Substructure Revealed by Genome-Wide Single Nucleotide Polymorphism Data
Source: PLoS One. 2011 Feb 9;6(2):e16747. doi: 10.1371/journal.pone.0016747 (PMC3036708; doi:10.1371/journal.pone.0016747)

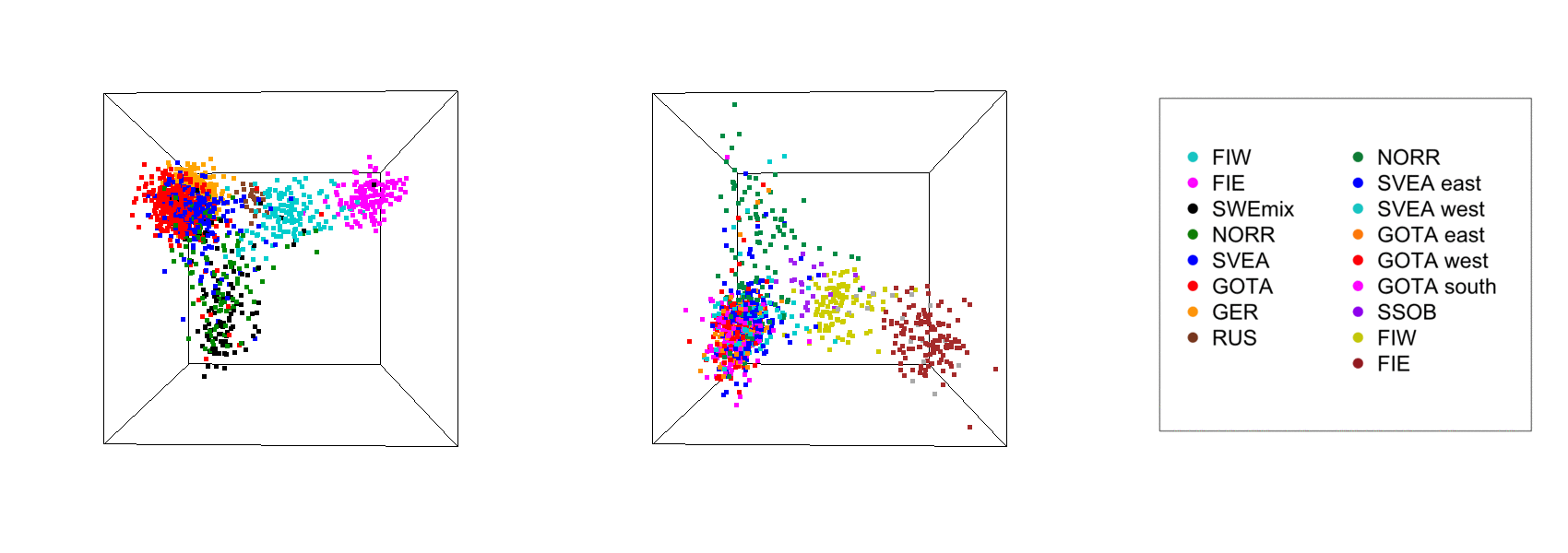

Supplement: Figure S1 — Multidimensional scaling plots of genetic distances between individuals in three dimensions. Identity by state distances in Northern Europe (left), and Sweden and Finland (right). The proportions of variance explained by the three axes are 0.64%, 0.24%, and 0.17% in Northern Europe and 1.04%, 0.26%, and 0.24% in Sweden and Finland, respectively. The animation files can be opened e.g. in most internet browsers. Abbreviations as in Table 1 and Table S1. (GIF) [file pone.0016747.s001.gif]

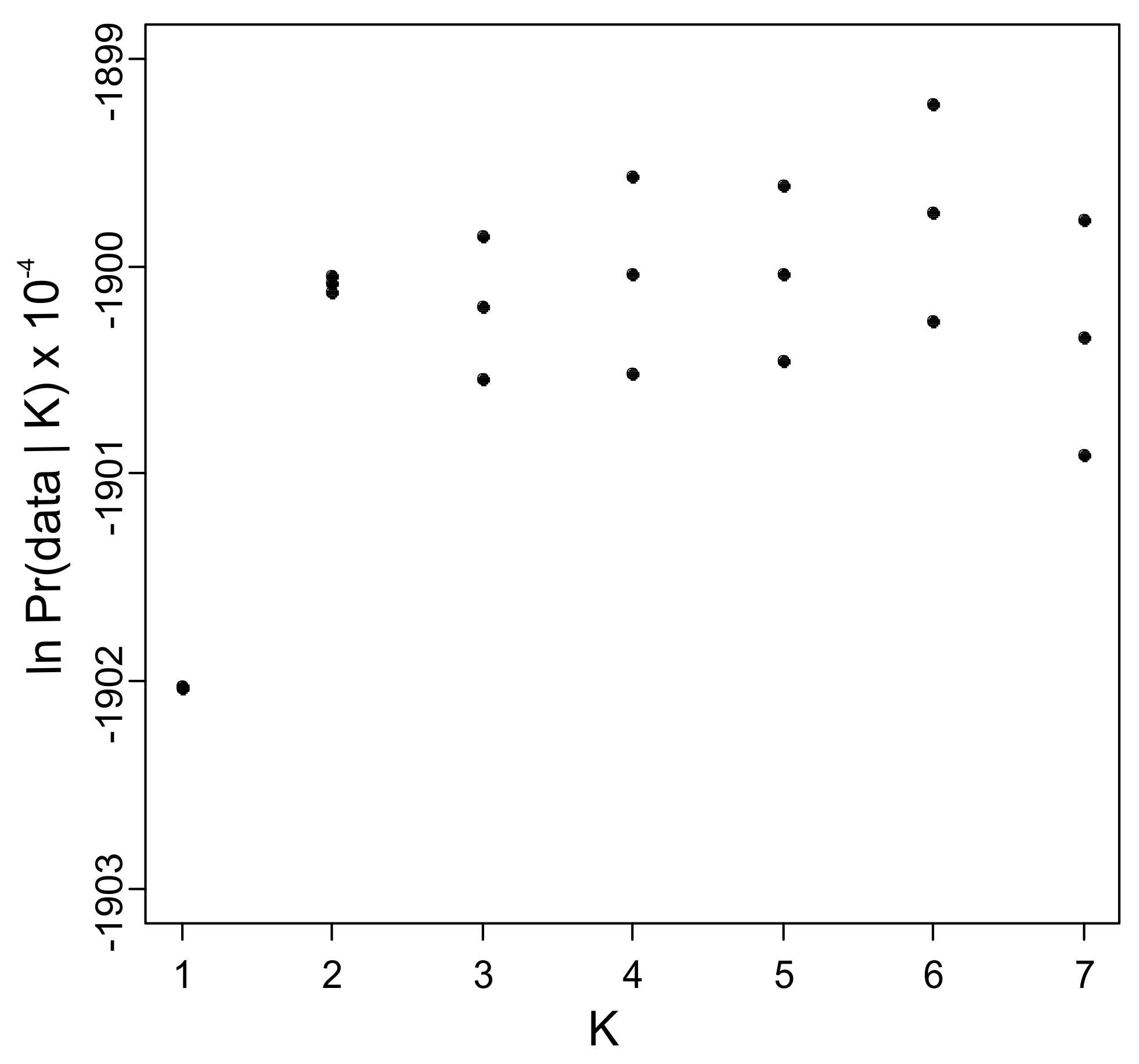

Supplement: Figure S2 — The probabilities of different numbers of clusters (K) in the Structure analysis of Northern Europe. (TIF) [file pone.0016747.s002.tif]

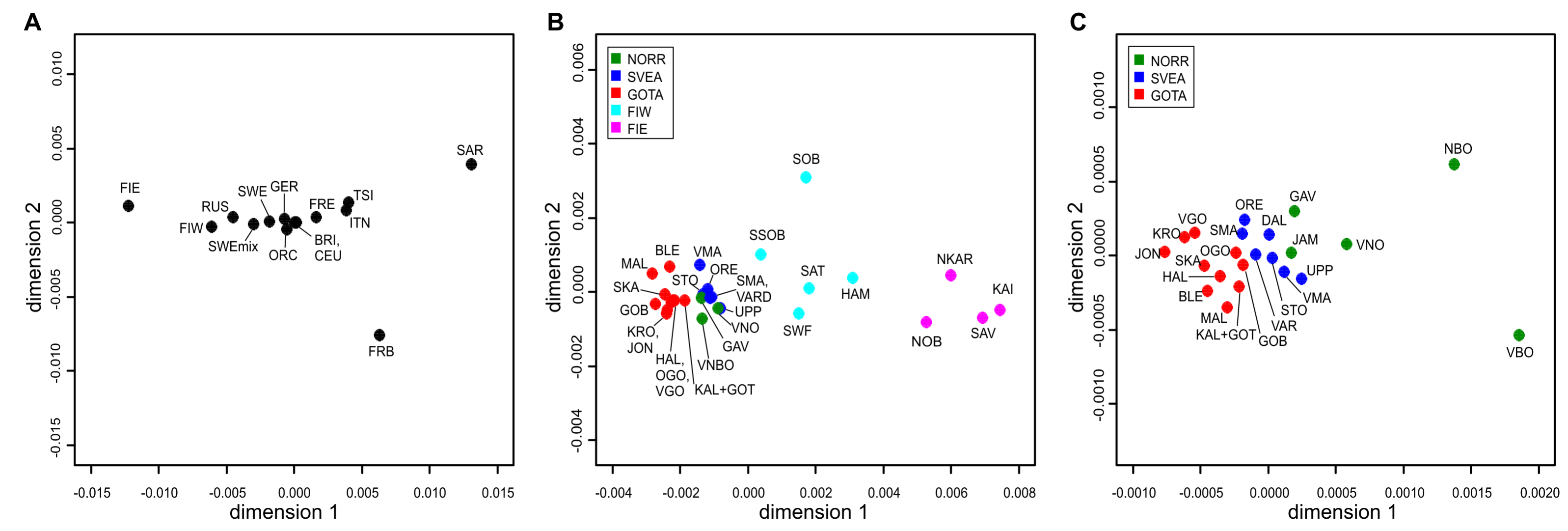

Supplement: Figure S3 — FST distances visualized by multidimensional scaling. Pairwise distances between European populations (a), Swedish and Finnish provinces (b), and Swedish provinces (c). The corresponding FST values can be found in Tables S2-S4. Abbreviations: Toscans in Italy (TSI) from HapMap; French (FRE), French Basque (FRB), North Italian (ITN), Orcadian (ORC), and Sardinian (SAR) from HGDP; Swedes with geographical information (NORR+SVEA+GOTA) (SWE); other abbreviations as in Table 1 and Table S1. (TIF) [file pone.0016747.s003.tif]

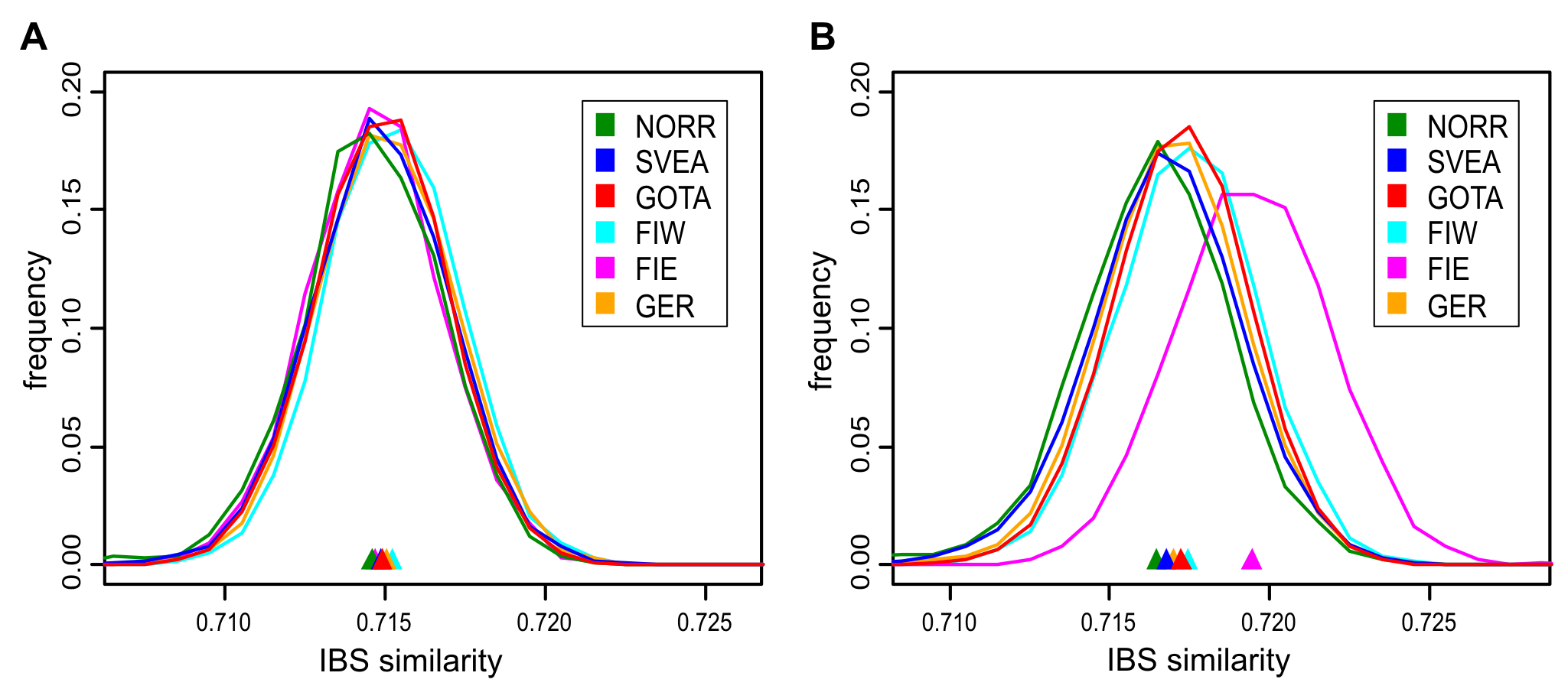

Supplement: Figure S4 — Distributions of pairwise identities by state (IBS). IBS distributions between six populations and Russians (a) and within the six populations (b). The location of distribution medians is denoted by triangles of corresponding color. In (a), Western Finland differed significantly from all other populations except Germany, and Germany from Norrland and Eastern Finland (p < 0.031 after a Bonferroni correction). In (b), Eastern Finland differed significantly from all other populations, Norrland from Götaland and Western Finland, and Western Finland from Svealand (p < 0.034 after a Bonferroni correction). Abbreviations as in Table 1. (TIF) [file pone.0016747.s004.tif]

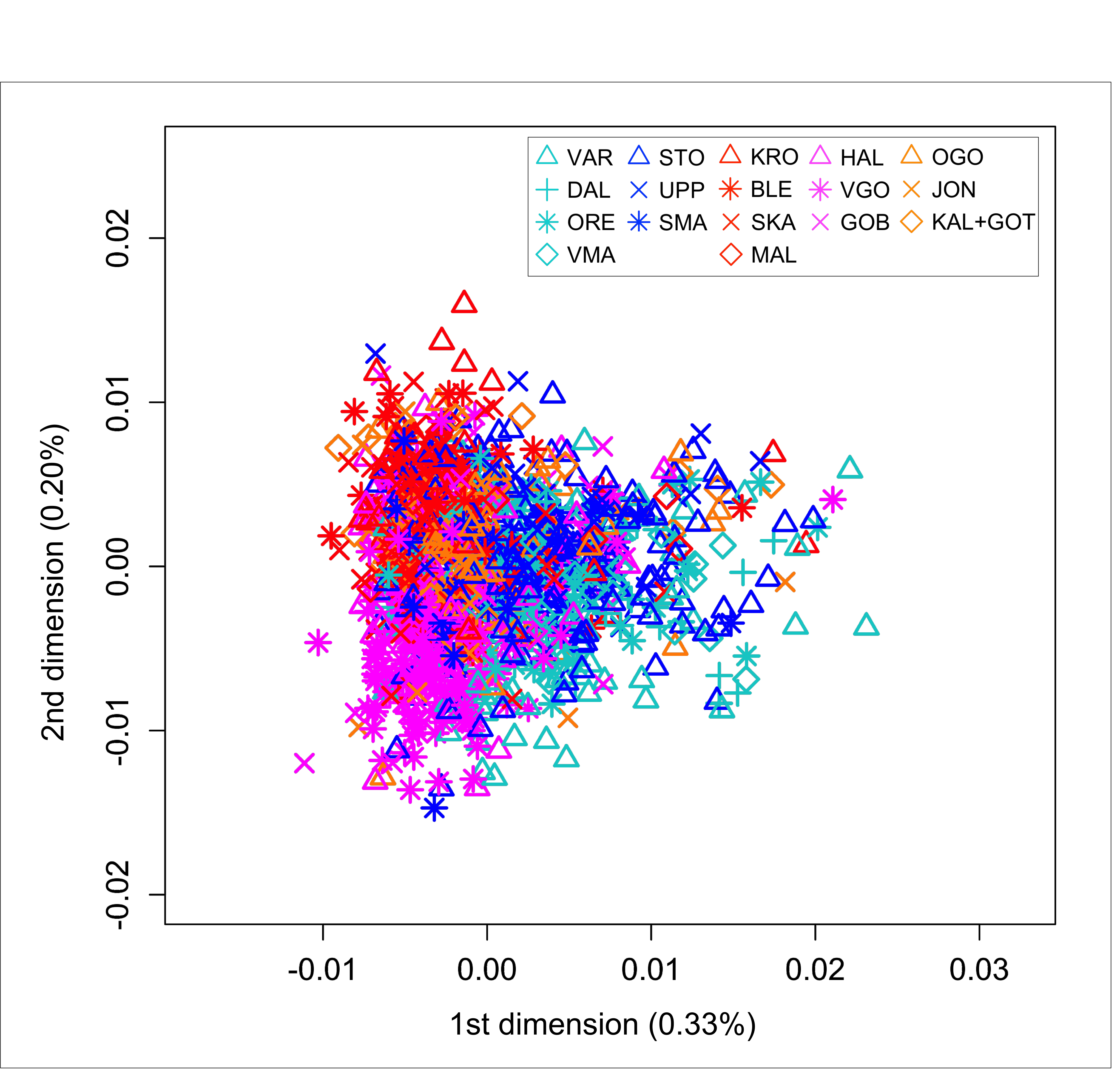

Supplement: Figure S5 — Multidimensional scaling plots of genetic distances between individuals in Svealand and Götaland. The genetic distance used is based on identity by state (IBS). Abbreviations as in Table S1. (TIF) [file pone.0016747.s005.tif]

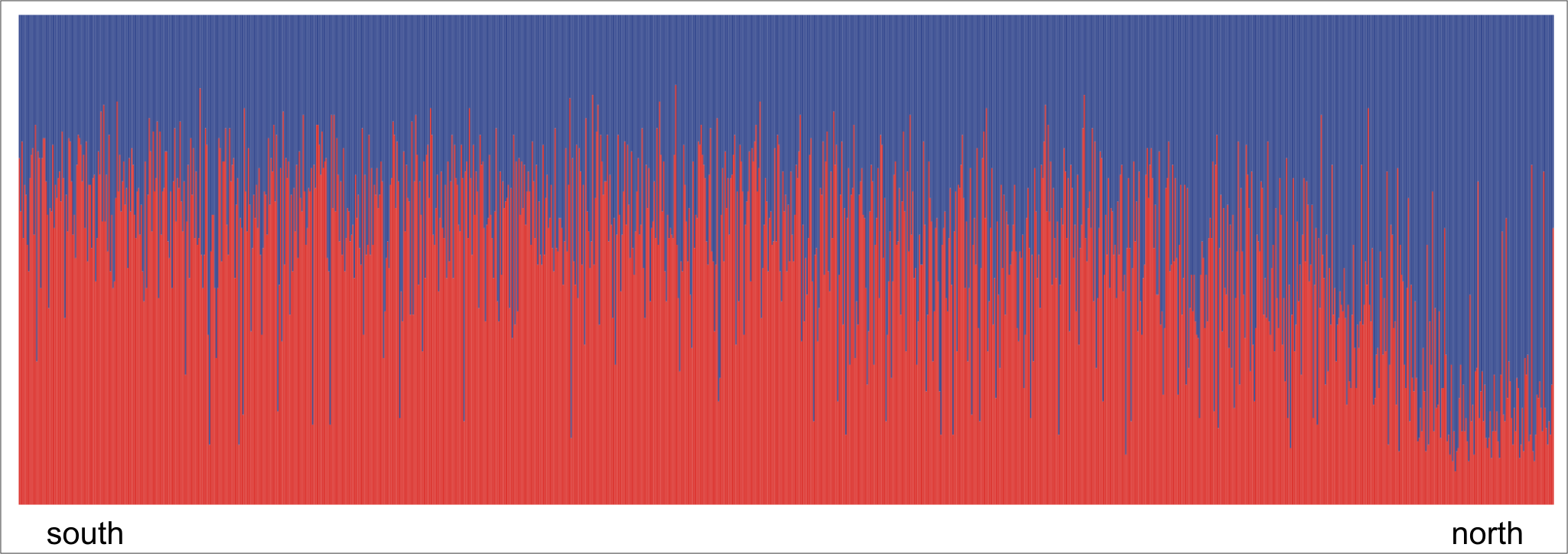

Supplement: Figure S6 — Clustering of Swedish individuals by the Structure software. Each individual is represented by a thin vertical line, and its proportional ancestry in the two inferred clusters is denoted by colors. The individuals are sorted according to latitude from south to north. (TIF) [file pone.0016747.s006.tif]

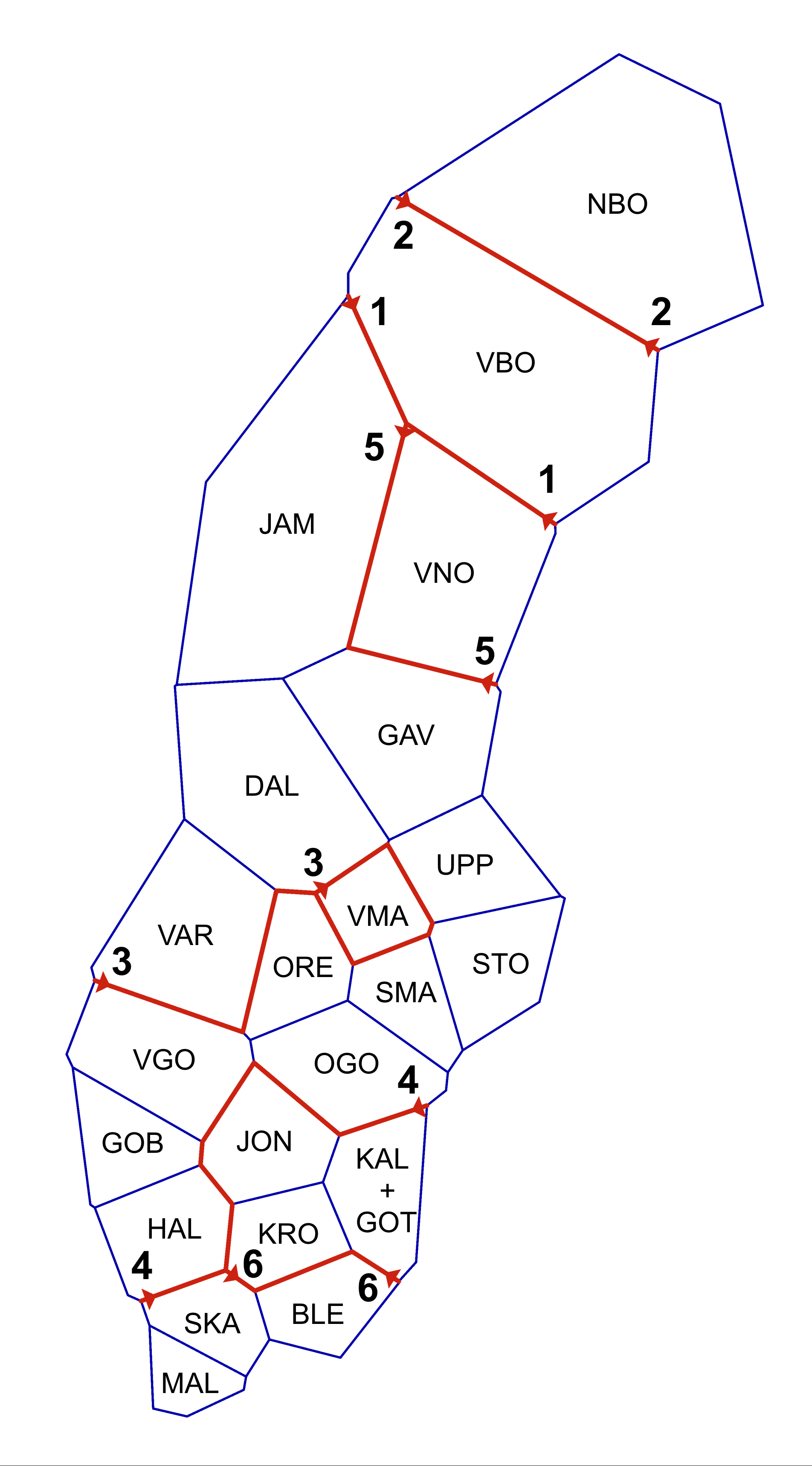

Supplement: Figure S7 — Six zones of strong genetic change inferred by the Barrier software. The borders (in red) are numbered in decreasing order of strength, and they are based on FST distances that have been corrected for the geographical distance between provinces. Note that the FST values differ significantly from zero only for the first two borders. (TIF) [file pone.0016747.s007.tif]

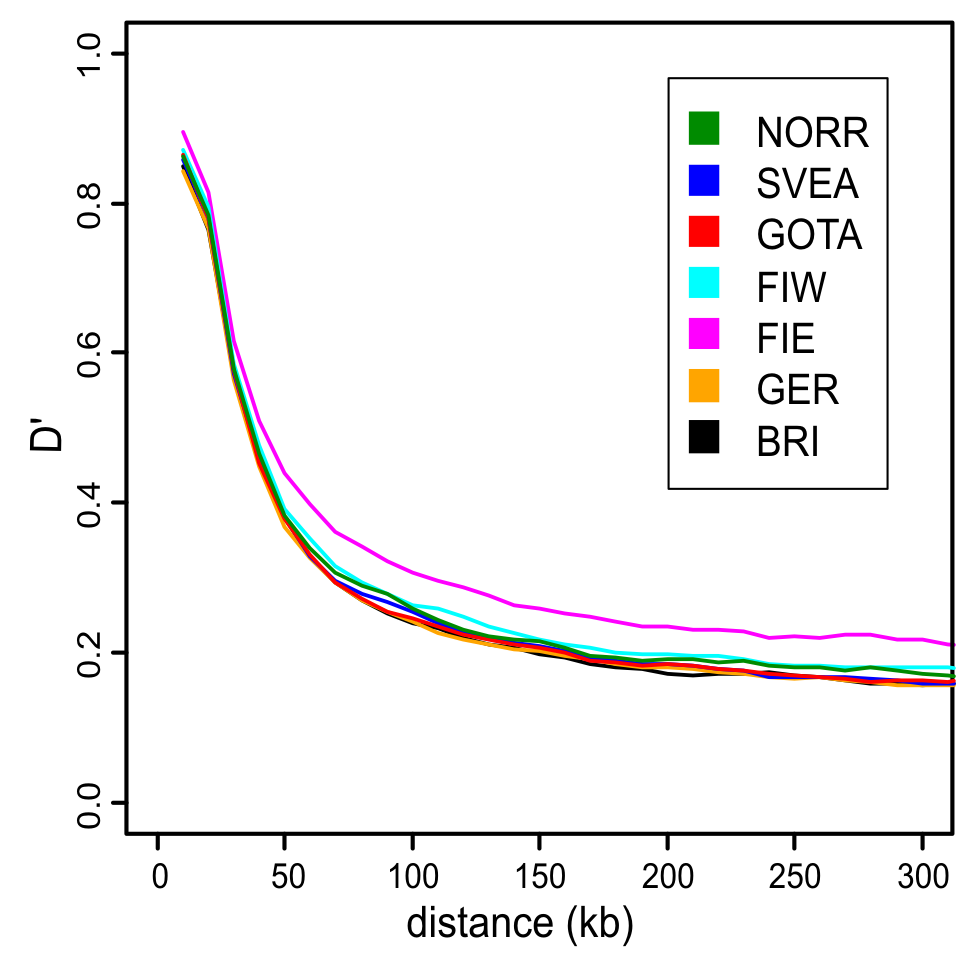

Supplement: Figure S8 — Linkage disequilibrium as a function of distance between markers. Median D' in overlapping 40 kb windows at 10 kb intervals is plotted for each population using 67620 marker pairs. All distributions differed significantly (p < 0.002) except Germany vs. Great Britain and Svealand vs. Götaland. Abbreviations as in Table 1. (TIF) [file pone.0016747.s008.tif]

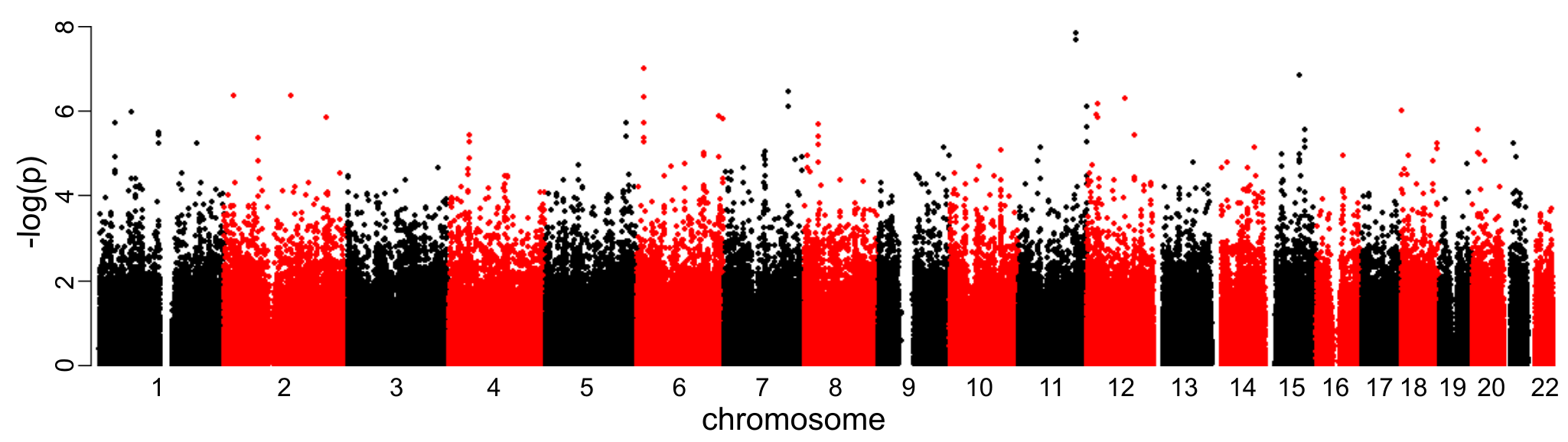

Supplement: Figure S9 — Genomic locations of SNPs whose allele frequency differs in Norrland. The p value from a chi-square test of allele frequencies between Norrland (n = 115) and the rest of Sweden (n = 635) is indicated for each SNP. The most differing SNPs are listed in Table S6 and Table S7. (TIF) [file pone.0016747.s009.tif]

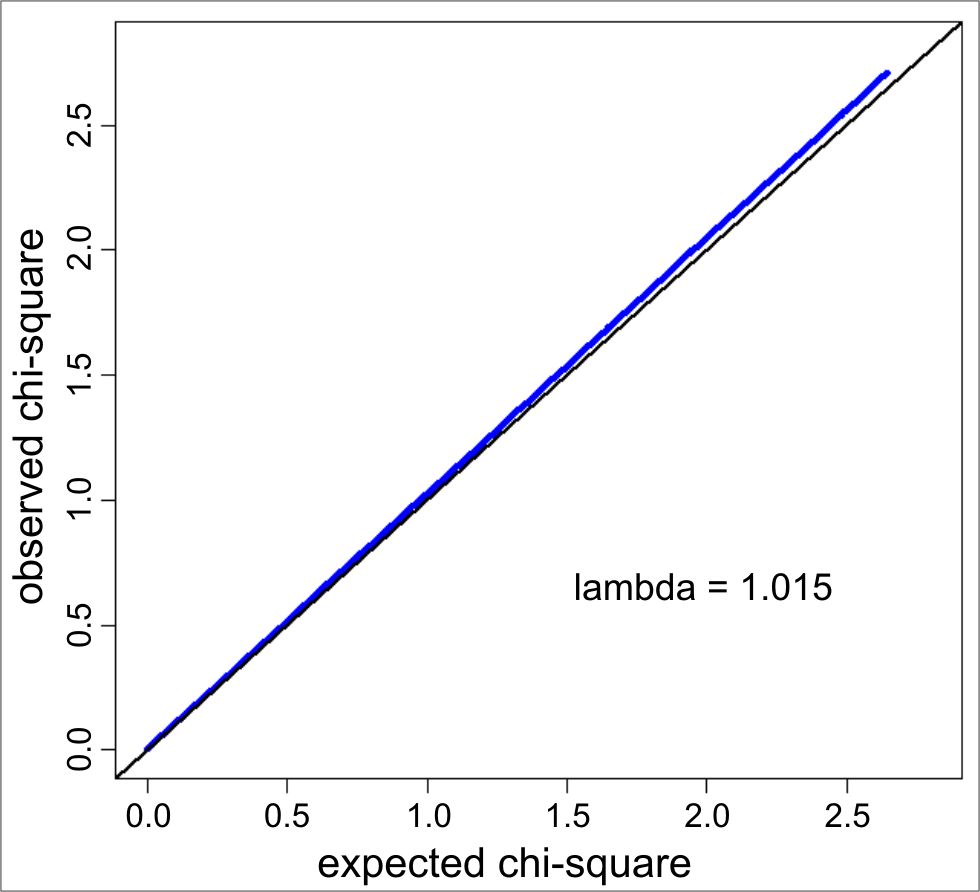

Supplement: Figure S10 — Differences between cases and controls in Dataset 3. A quantile-quantile plot of observed vs. expected test statistics (in blue) from a chi-square test of allele frequency differences between cases and controls for the SNPs that remain in Dataset 3 after quality control. Lambda denotes the overdispersion factor of observed vs. expected chi-square statistics. (TIF) [file pone.0016747.s010.tif]
